# Fertility in Norwegian testicular cancer patients

**DOI:** 10.1054/bjoc.1999.0989

**Published:** 2000-01-18

**Authors:** S D Fosså, Ø Kravdal

**Affiliations:** The Norwegian Radium Hospital, Montebello, Oslo, N-0310, Norway; The Norwegian Cancer Registry and Department of Economics, Section for Demography, University of Oslo, PO Box 1095 Blindern, Oslo, N-0317, Norway

**Keywords:** fecundity, incidence, infecundity, infertility, parity, treatment

## Abstract

The intention was to explore the relationship between fertility and testicular cancer, including the possibly treatment-induced changes over time in the post-diagnostic fertility. Data are from the Norwegian Cancer Registry, The Norwegian Population Register and the Population Censuses. By estimating Poisson regression models, birth rates among testicular cancer patients were compared with those of other men who had the same age, parity and duration since previous birth. Poisson regression models were also estimated to check whether men's parity has an effect on the cancer incidence. Fertility rates among testicular cancer patients born after 1935 and treated before 1991 decreased by roughly 30% when compared with the normal population. The introduction of cisplatin chemotherapy and of nerve-sparing RPLND in the 1980s seems to have enabled more patients with non-seminoma to father a child after treatment, or at least shortened the time to conception. Moreover, the risk of being diagnosed with seminoma is reduced with increasing parity. This suggests that the relatively low fertility after diagnosis may be partly due to the continuing inherent influence of a sub- or infecundity that also had a bearing on the development of the disease. © 2000 Cancer Research Campaign


British Journal of Cancer (2000) **82**(3), 737–741
© 2000 Cancer Research Campaign
DOI: 10.1054/ bjoc.1999.0989, available online at http://www.idealibrary.com on IDEAL®

### Fertility in Norwegian testicular cancer patients

**SD Fosså[1] and Ø Kravdal[2]**

[1]The Norwegian Radium Hospital, Montebello, N-0310 Oslo, Norway; [2]The Norwegian Cancer Registry and Department of Economics, Section for Demography, University of Oslo, PO Box 1095 Blindern, N-0317 Oslo, Norway

**Summary** The intention was to explore the relationship between fertility and testicular cancer, including the possibly treatment-induced changes over time in the post-diagnostic fertility. Data are from the Norwegian Cancer Registry, The Norwegian Population Register and the Population Censuses. By estimating Poisson regression models, birth rates among testicular cancer patients were compared with those of other men who had the same age, parity and duration since previous birth. Poisson regression models were also estimated to check whether men's parity has an effect on the cancer incidence. Fertility rates among testicular cancer patients born after 1935 and treated before 1991 decreased by roughly 30% when compared with the normal population. The introduction of cisplatin chemotherapy and of nerve-sparing RPLND in the 1980s seems to have enabled more patients with non-seminoma to father a child after treatment, or at least shortened the time to conception. Moreover, the risk of being diagnosed with seminoma is reduced with increasing parity. This suggests that the relatively low fertility after diagnosis may be partly due to the continuing inherent influence of a sub- or infecundity that also had a bearing on the development of the disease. © 2000 Cancer Research Campaign

**Keywords:** fecundity; incidence; infecundity; infertility; parity; treatment

In Norway, the standardized incidence of testicular cancer has increased from 2.7 per 100 000 in 1955 to 8.5 per 100 000 in 1992, with little difference between seminoma and non-seminoma (Wanderaas et al, 1995). The mean age of seminoma and non-seminoma patients at the time of diagnosis is 40 and 32 years respectively. When confronted with such a diagnosis, most of these young men ask their responsible physician about their chances of having children after treatment. Though many mono-institutional studies have demonstrated that long-term sperm cell production and ejaculation are preserved in the majority of patients treated during the last decade (Petersen et al, 1998; Jacobsen et al, 1999), population-based studies on post-treatment fertility are rare.

From clinical praxis it has been suspected for a long time that there are links between sub- or infertility and the development of testicular cancer (Giwermann and Petersen, 1998). In 1994, the United Kingdom Testicular Cancer Study Group described a significant association between low fertility or sterility and the risk of being diagnosed with testicular cancer. Møller and Skakkebæk (1999) demonstrated that paternity by itself and increasing parity were associated with a lower risk of testicular cancer.

The objective of this study was to check whether Norwegian testicular cancer patients had fewer children before diagnosis than men of the same age without this disease and, more importantly, whether their fertility after diagnosis differed from that of others. Stage and histology, and thereby largely the treatment modality (radiotherapy, chemotherapy, retroperitoneal surgery), were taken into account. Also, the changes over time were assessed.

*Received 13 May 1999*
*Revised 20 July 1999*
*Accepted 23 August 1999*

*Correspondence to*: SD Fosså

### MATERIALS AND METHODS

The analysis was based on individual sociodemographic life histories up to the end of 1991 for all men with a Norwegian personal identification number (given to everyone who has lived in Norway for some time after 1960) who are born after 1935. These 'life histories' were extracted from the Norwegian Population Register and the Population Censuses of 1960, 1970 and 1980, and included information about date of death and emigration, date of birth for all children the man fathered up to 1991, marital status, education and various other socioeconomic characteristics at the time of the censuses. The data were based on a social definition of parenthood, i.e. the fathers were linked with their social rather than biological offspring.

These life histories were matched with data from the Norwegian Cancer Registry, which from 1953 has received information on all cancer cases in the country. This compulsory reporting system is based on pathology and cytology reports, clinical records and death certificates, and provides information about site, basis for the diagnosis, histological grade and type, and the stage of the disease at the time of diagnosis. The matching of the data was approved by the Norwegian Data Inspectorate.

The multiplicative Poisson regression model

(1) $f = \exp(bx)\exp(cy)$

was estimated for the birth intensity f. x is a vector of sociodemographic covariates age, period, parity, duration since previous birth, marital status and educational level. These covariates are all categorical and time-varying. A level for each covariate is defined for each month during the follow-up period, and refers to the situation at that time (age, period, parity, duration) or that in the last previous census (education, marital status).

The variable y is a categorical time-varying disease indicator with one level up to the diagnosis of a testicular cancer, if any, and 3–5 levels afterwards, defined as a combination of stage and duration since diagnosis. In these data, stage is defined as 'localized' (non-metastatic, stage I), 'regional' (spread to regional lymph nodes, stage II), haematogeneous 'distant' spread (i.e. to parenchymatous organs or to non-regional lymph nodes, stage III) or unknown (only 1%). Because of the small size of the latter category, it is combined with localized. The effect vectors are b and c.

The men were followed from age 17 and censored at the time of emigration, death, or the end of 1991. In principle, all parity transitions can be considered in such an analysis (up to 18, which is the highest parity reached in these data), but for practical reasons a limit was set. It was decided to censor at the birth of the third child, because few Norwegian men have more than three children (Kravdal, 1994).

For cancer patients, censoring was done at the time of the first birth after diagnosis. This is because fertility among those who have already proved their fertility by having one child after diagnosis is much less interesting. In other words, c is a measure of how the chance of having child number $n + 1$ differs between two groups of men who currently have had $n$ children, the same duration since last birth, the same age and also the same other observed sociodemographic characteristics. One group comprises those who had a testicular cancer and had all their children before diagnosis; the other group comprised those without such a diagnosis.

Separate models were estimated for seminoma and non-seminoma, and for patients diagnosed before 1980 and thereafter. This cut-off point was chosen because of the important treatment changes initiated at that time (Fosså et al, 1991). The following is a brief summary of these changes.

Up to 1980, all patients with stage I disease were treated by abdominal radiotherapy (target dose 40–50 Gy). Stage II patients received the same radiotherapy in addition to mediastinal irradiation. Patients with distant metastases or with recurrent malignancy were treated by available chemotherapy (without cisplatin).

From 1980, non-seminoma stage I patients underwent retroperitoneal lymph node dissection (RPLND). After 1988, such patients were included in a surveillance policy. Metastatic or recurrent patients were given cisplatin-based combination chemotherapy followed by surgery. In stage I seminoma patients, the target dose to the para-aortic lymph nodes was reduced from 40 Gy to 30 Gy. Metastatic seminoma was treated with cisplatin-based chemotherapy and small-field irradiation.

Five-year relative survival rates increased from 61% in the late 1960s to 93% in the late 1980s (Cancer Registry, 1993).

As a supplementary and simple description of the impact of testicular cancer on fertility, the probabilities of having had a first child were calculated for different ages for persons who were childless at, say, age 20. If A is the integral of the first-birth rates from age 20 up to the age in focus, such a probability $P$ (also denoted as a 'partial' probability, because it is based on birth rates exclusively, thus disregarding the chances of not surviving up to that age) is given by

(2) $P = 1 - \exp(-A)$

Constant birth rates are assumed for 1-year intervals, and are estimated by dividing the number of births in each interval by the corresponding exposure time.

In addition, the multiplicative Poisson regression model

(3) $i = \exp(dz)$

was estimated for the cancer incidence i. z is a vector of sociodemographic covariates age, period, marital status, educational level (defined as in the fertility model) and parity. Parity was defined for each month of follow-up and referred to the total number of children the man had fathered up to that time. In other words, it was estimated how the risk of being diagnosed with testicular cancer at a given age was related to parity at that age, net of differences in period, education and marital status. The men were followed from age 17 up to time of emigration, death or the end of 1991. This method was also used with these data in several studies of other cancer types (Kravdal, 1995; Harvei and Kravdal, 1997).

The models were estimated in the AMFIT module in the EPICURE program system (Preston et al, 1993). A self-made program (in the PASCAL language), operating on the individual-level register and census data, was used to compute the multi-dimensional tables of events (number of births or cancer cases) and exposures that were fed into AMFIT.

### RESULTS

During the period under study, seminoma and non-seminoma patients fathered 171 and 250 children, respectively, within 10 years after diagnosis (Table 1). In comparison, there were 1.3 million births among other men (not shown).

**Table 1** Fertility rates for Norwegian men with a testicular cancer diagnosis fewer than 10 years previously, relative to men without such a diagnosis (with 95% confidence intervals)[a]

| | Non-seminoma | | | Seminoma | | |
|---|---|---|---|---|---|---|
| | Estimates | | *n* | Estimates | | *n* |
| Men without testicular cancer[b] | 1.0 | | | 1.0 | | |
| Men with testicularcancer diagnosed fewer than 10 years previously | | | | | | |
| Local | 0.78[c] | (0.67–0.91) | 172 | 0.69[c] | (0.59–0.81) | 147 |
| Regional | 0.45[c] | (0.34–0.60) | 48 | 0.14[c] | (0.03–0.55) | 19 |
| Distant | 0.33[c] | (0.23–0.47) | 30 | 0.35[c] | (0.18–0.67) | 5 |

[a]Only the effects of the disease variable are shown in the Table, but also age (15 levels), period (4 levels), parity (0, 1, or 2), duration since last previous birth (7 levels), education (4 levels) and marital status (4 levels) were included in the model. It was censored 10 years after diagnosis, if any. [b]Reference category. [c]Significantly different from 1 at the 5% level. *n,* number of births among men in this category.

 *© 2000 Cancer Research Campaign*

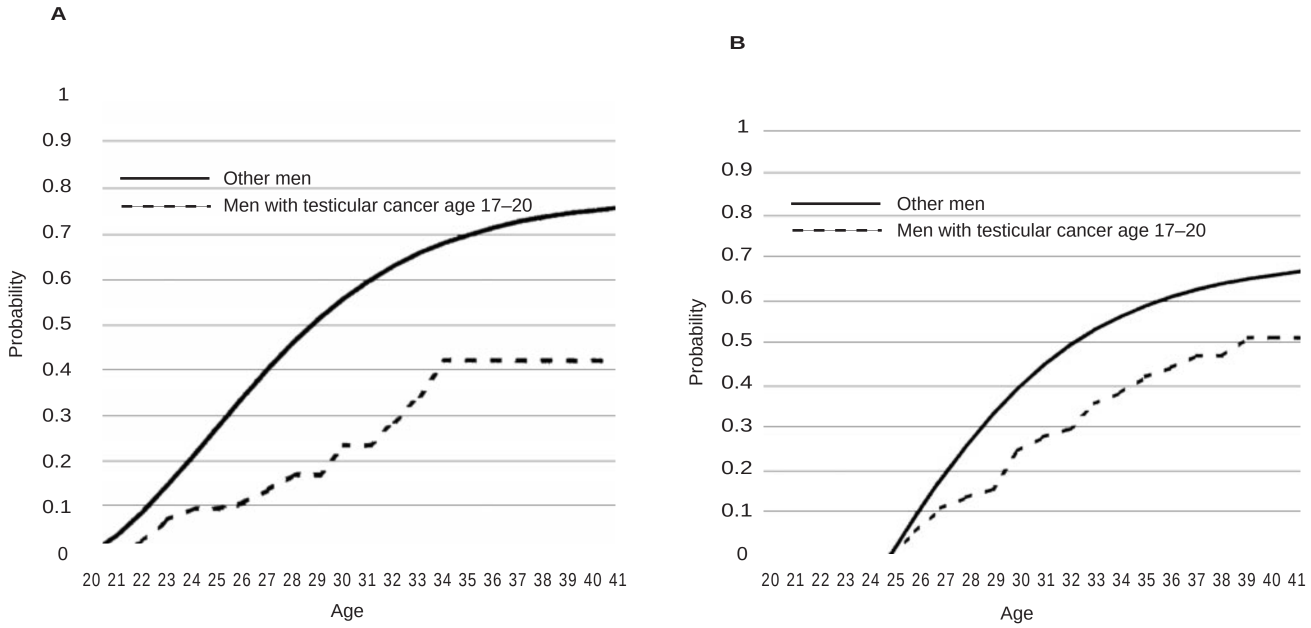

**Figure 1** (**A**) Probabilities of having had a first child, by age, among Norwegian men with or without a testicular cancer diagnosis who were childless at age 20. (**B**) Probabilities of having had a first child, by age, among Norwegian men with or without a testicular cancer diagnosis who were childless at age 25

**Table 2** Fertility rates for Norwegian men with a testicular cancer diagnosis, relative to men without such a diagnosis (with 95% confidence intervals)[a]

| | Before 1980 | | | After 1980 | | |
|---|---|---|---|---|---|---|
| | Estimates | | *n* | Estimates | | *n* |
| *Non-seminoma* | | | | | | |
| Men without tesitcular cancer[b] | 1.0 | | | 1.0 | | |
| Men with testicular cancer diagnosed fewer than 5 years previously | | | | | | |
| Local | 0.72[c] | (0.56–0.94) | 58 | 0.85 | (0.67–1.06) | 78 |
| Regional/distant | 0.25[c] | (0.13–0.48) | 9 | 0.49[c] | (0.36–0.64) | 55 |
| Men with testicular cancer diagnosed 5–10 years previously | | | | | | |
| Local | 1.08 | (0.64–1.59) | 17 | 0.57[c] | (0.37–0.90) | 19 |
| Regional/distant | 0.45 | (0.11–1.82) | 2 | 0.26[c] | (0.15–0.46) | 12 |
| Men with testicular cancer diagnosed more than 10 years previously | | | | | | |
| Local | | | | | | |
| Regional/distant | 0.65 | (0.37–1.18) | 11 | 0.71[c] | (0.53–0.97) | 41 |
| *Seminoma* | | | | | | |
| Men without testcular cancer[b] | 1.0 | | | 1.0 | | |
| Men with testicular cancer diagnosed fewer than 5 years previously | | | | | | |
| Local | 0.77 | (0.58–1.02) | 49 | 0.61[c] | (0.49–0.77) | 72 |
| Regional/distant | 0.41[c] | (0.17–0.99) | 5 | 0.49[c] | (0.29–0.85) | 13 |
| Men with testicular cancer diagnosed 5–10 years previously | | | | | | |
| Local | 0.63 | (0.24–1.41) | 6 | 0.75 | (0.48–1.16) | 20 |
| Regional/distant | 0.40 | (0.06–2.86) | 1 | 0.56 | (0.23–1.35) | 5 |
| Men with testicular cancer diagnosed more than 10 years previously | | | | | | |
| Local | | | | | | |
| Regional/distant | 0.47 | (0.15–1.46) | 3 | 0.86 | (0.55–1.34) | 19 |

[a]Only the effects of the disease variable are shown in the Table, but also age, period, parity, duration since last previous birth, education and marital status were included in the model. The categories are as described in note[a] to Table 1. [b]Reference category. [c]Significantly different from 1 at the 5% level. *n,* number of births among men in this category.

*© 2000 Cancer Research Campaign*

**Table 3** Effects of parity on the risk of being diagnosed with testicular cancer (with 95% confidence intervals)[a]

| | Non-seminoma | | | Seminoma | | |
|---|---|---|---|---|---|---|
| | | Estimates | *n* | | Estimates | *n* |
| 0 child[b] | 1.00 | | 596 | 1.00 | | 379 |
| 1 child | 1.03 | (0.87–1.21) | 213 | 0.76[c] | (0.63–0.92) | 163 |
| 2 children | 0.97 | (0.81–1.16) | 233 | 0.78[c] | (0.66–0.92) | 289 |
| 3 children | 0.83 | (0.64–1.08) | 76 | 0.60[c] | (0.47–0.75) | 104 |
| 4 or more children | 1.10 | (0.75–1.62) | 31 | 0.80 | (0.58–1.09) | 50 |

[a]Only the effects of parity are shown in the Table, but also age, education are marital status were included in the model. The categories are as described in note [a] to Table 1. [b]Reference category. [c]Significantly different from 1 at the 5% level. *n*, number of diagnoses among men in this category.

In the regression estimates presented, only the relation between fertility and the disease variable is diplayed (Table 1) (controls for education and marital status were included, but were not important). Having a localized cancer reduced fertility by about 30% compared to the normal population, while a stronger reduction was seen among men whose cancer had spread at the time of diagnosis. These estimates are for the entire 10-year period after diagnosis. The deviation from normal fertility was slightly more pronounced during the first few years after diagnosis (not shown).

As a simple illustration, the probabilities of having had a first child within different ages are plotted in Figure 1 for men born 1945–1965. In Figure 1A, probabilities are shown for two groups of men who were still childless at age 20. One group includes about 100 men who were diagnosed with testicular cancer at age 17–20 (regardless of stage and histology), and the other group includes all other men. In the latter group, 76% had a child when they were 41 years old, whereas the corresponding proportion among the testicular cancer patients was only 42%. Similar probabilities for two groups of men who were still childless at age 25 are plotted in Figure 1B. One group includes about 250 men with a testicular cancer diagnosis at age 17–25, and the other group includes all other men.

Separate regression models were estimated for periods before and after 1980 (Table 2). For non-seminoma, there were quite strong indications of an increasing relative fertility over time when it was focused on men who had been diagnosed with metastasis less than 5 years previously. Before 1980, men in this situation only had nine children, whereas the corresponding number for the later period was 55. These differences reflect, of course, both fertility rates and the number of men under exposure for births, which in turn was determined by testicular cancer incidence as well as survival. The fertility rates were estimated to have doubled, from one-quarter of the level among other men before 1980, to one-half in the later period. The confidence intervals barely overlapped. With respect to fertility 5–10 years after diagnosis, there were indications, albeit weaker, of an opposite trend over time. For seminoma, there were very modest differences in relative fertility between the periods before and after 1980.

The risk of developing seminoma depended significantly on parity. For example, the risk for a man with three children was 40% lower than that for a childless, but otherwise similar, man. The risk for a four-child father was not significantly reduced, but the group was quite small. When pooled together, a relative risk of 0.67, significantly different from 1, appeared for those with three or more children (Table 3). On the other hand, there was no association between parity and the non-seminoma incidence.

### DISCUSSION

The birth rates in this study reflect almost exclusively the in vivo biological fertility. Adoptions are very rare in Norway, and assisted fertilization also counts very little. Furthermore, the few children born fewer than 9 months after their father's diagnosis of testicular cancer are considered as 'post-treatment', although their conceptions most probably had taken place prior to the diagnosis and treatment.

Our strategy of censoring at the time of a first birth after diagnosis was not critical for the results. Also, those who already had a child after diagnosis, and thus signal an ability to conceive, displayed a subsequent fertility lower than that of men at the same parity level without the testicular cancer diagnosis. This deficit was similar to that for the first birth after diagnosis.

Testicular cancer was found to be associated with relatively low fertility before diagnosis. Seminoma patients, but not those with non-seminoma, had significantly fewer children at the time of diagnosis than men with otherwise similar socio-demographic characteristics. We thus confirm the results of the two former comparable epidemiological studies (United Kingdom Testicular Cancer Study Group, 1994; Möller and Shakkebæk, 1999). The most plausible explanation for the relationship between (prediagnostic) subfertility and the seminoma incidence is that some types of primary hypogonadism and seminoma may share some aetiological factors during early embryonal life, leading to disturbed differentiation of primordial cells – the cells from which the male gonads develop. This disturbance may be expressed as infecundity, reduced spermatogenesis, and may even contribute to the development of testicular cancer, in particular seminoma, affecting one or both of the testicles. The fact that parity effects are less pronounced in non-seminoma patients could perhaps be partly due to their generally lower age. At a relatively low age, low parity is more a signal of choice, while it is more likely to indicate physiological limitations at a higher age.

A clinical implication of this result is that especially the childless older seminoma patients should undergo treatment which is as fertility-saving as possible in order to allow a maximum recovery of the spermatogenesis. These arguments strongly favour the application of a wait-and-see policy in patients who want to father a child after diagnosis (Warde et al, 1993).

In addition to a low prediagnostic fertility for seminoma patients, birth rates were low also after a testicular cancer diagnosis compared to those of other men at the same age and parity in the same period. Among men with localized cancers, of either histological type, the birth rates after diagnosis were about

 *© 2000 Cancer Research Campaign*

one-quarter lower than in the remaining population, while the gap was more than twice as large in cases of metastasized cancers.

There are several possible reasons for this lowered fertility after diagnosis and treatment. One reason, which is relevant only for the seminoma patients, is a continuing influence of an inherent sub- or infecundity that also existed before diagnosis. Another reason is a reduced desire for more children after an exhausting treatment for a life-threatening malignancy. A perceived risk of malformations of the offspring due to prior cytotoxic therapy of the father may also contribute to weaken fertility desires and may lead not only to postponement but also rejection of further childbearing. Any such voluntary postponement of post-treatment fatherhood is, of course, of greater significance for the generally older seminoma patients (and their partners) than for those with non-seminoma.

Possibly the most important reason for the low post-diagnostic fertility among testicular cancer patients is the treatment. The use of abdomino-pelvic radiotherapy and cytostatics leads to decreased spermatogenesis, which, depending on the type of treatment and cumulative doses and the patient's age, may or may not recover. Secondly, traditional RPLND, performed in many non-seminoma patients before 1985 with the complete resection of sympathetic nerve fibres, leads to 'dry ejaculation' and thus to infecundity. The change of treatment modalities after 1980 is expected to have reduced the risk of treatment-induced infecundity for patients treated after 1980 (Petersen et al, 1998; Jacobsen et al, 1999). The Registry data support the expectations, and demonstrate that this effect is most pronounced in metastatic non-seminoma patients, though the picture is not entirely consistent. When the focus is on the first 5 years after diagnosis, there are indications of improvement in fertility for the observation period 1980–1991. On the other hand, there are also weak indications of a gradually larger fertility deficit during recent years among men diagnosed with testicular cancer 5–10 years previously. This might be explained by a changing force of selection: with current standard chemotherapy, spermatogenesis recovers after 2–3 years, whereas the recovery took place later, if ever, in the 1960s and 1970s. The patients treated after 1980 who had still not had a child 5 years after diagnosis, in spite of the improved therapy, may to a much larger extent than before comprise a group of persons with treatment-independent infecundity problems or weak fertility desires. The favourable time trend will probably continue among non-seminoma patients as a result of the surveillance policy and nerve-sparing RPLND since 1989. In seminoma patients, improvement of fertility will presumably be less pronounced, as long as changes in the treatment remain more limited.

In summary, this study shows, first, that the fertility rates among Norwegian testicular cancer patients born after 1935 and treated before 1991 decreased by roughly 30% when compared with the normal population. Secondly, there are quite strong indications that the introduction of cisplatin-based chemotherapy and limited or nerve-sparing RPLND in the 1980s made it possible for more non-seminoma patients to have (another) child after diagnosis, or at least to have a child earlier. Thirdly, the risk of being diagnosed with seminoma is reduced with increasing parity. This suggests that the relatively low fertility after diagnosis may be partly due to the continuing influence of sub- or infecundity problems that also had a bearing on the development of the disease. Consequently, introduction of a wait-and-see policy for childless patients with stage I seminoma may be advantageous in an attempt to preserve pre-existing (though low) fertility as much as possible.

### ACKNOWLEDGEMENTS

Thanks are due to Statistics Norway for allowing the use of the data.

### REFERENCES

Cancer Registry (1996) *Cancer in Norway 1993*. Norwegian Cancer Registry: Oslo

Fosså SD, Aass N, Ous S and Wæhre H (1991) Long-term morbidity in testicular cancer. *Scand J Urol Nephrol* **138:** 241–246

Giwercman A and Petersen PM (1998) Testicular cancer and gonodal function: biological and epidemiological aspects and effects of treatment. In: *Germ Cell Tumors IV*, Jones WG, Appleyard I, Harnden P and Joffe JK (eds). John Libbey: London

Harvei S and Kravdal Ø (1997) The importance of marital and socioeconomic status in incidence and survival of prostate cancer. *Prev Med* **26**: 623–632

Jacobsen KD, Ous S, Wæhre H, Trasti H, Stenwig AE, Lien HH, Aass N and Fosså SD (1999) Ejaculation in testicular cancer patients after post-chemotherapy retroperitoneal lymph node dissection. *Br J Cancer* **80**: 249–255

Kravdal Ø (1994) Components of the recent fertility increase in Norway: period and cohort perspectives. In: *Sociodemographic Studies of Fertility and Divorce in Norway with Emphasis on the Importance of Economic Factors*, Kravdal Ø (ed), SØS 90. Statistics Norway: Oslo

Kravdal Ø (1995) Is the relationship between childbearing and cancer incidence due to biology or lifestyle? Examples of the importance of using data on men. *Int J Epidemiol* **24**: 477–484

Møller H and Skakkebæk NE (1999) Risk of testicular cancer in subfertile men: case–control study. *Br Med J* **318**: 559–562

Petersen PM, Giwercman A, Skakkebæk NE and Rørth M (1998) Gonadal function in men with testicular cancer. *Semin Oncol* **25**: 224–233

Preston DL, Rubin JH, Pierce PA and McConney ME (1993) *Epicure User's Guide*. Hirosoft International Corporation: Seattle

United Kingdom Testicular Cancer Study Group (1994) Aetiology of testicular cancer: association with congenital abnormalities, age at puberty, infertility, and exercise. *Br Med J* **308**: 1393–1399

Wanderaas EH, Tretli S and Fosså SD (1995) Trends in incidence of testicular cancer in Norway 1955–1992. *Eur J Cancer* **31A**: 2044–2048

Warde PR, Gospodarowicz MK, Goodman PJ, Sturgeon JF, Jewett MA, Catton CN, Richmond H, Thomas GM, Duncan W and Munro AJ (1993) Results of a policy of surveillance in stage I testicular seminoma. *Int J Radiation Oncol Biol Phys* **27**: 11–15

© 2000 Cancer Research Campaign
